# 

**Published:** 1975

**Authors:** 


					W)
WM\
? 3P3 ? . - : ; ' ' : - ; - -
' j, . *?** ?% , v v*,'" *- ]j|g" ^cm.'1' & h (- *3^ f'-A ?>' ' V ^ ir? ?k ' " *r ^ ^ ?
'
5 N?Bwi './?<< {mm
SlllS&Si?
ggg ^ ^JjL:'
B&
?&
v;y .'?": :.
iM
'$"f! :?. "V ?
-? -'? r.v'--.'- ?J ? 0"? ' ? t ?" 1 ?*? ra
RISTOL
MEDICO
CHIRURGICAL
JOURNAL
? V
I -;?>
p/BV-"'' ' " m
of ^
JULY/OCTOBER 1975
VOL. 90 (lil/lv) No. 335/6

				

